# Intestinal parasitic infections and determinant factors among school-age children in Ethiopia: a cross-sectional study

**DOI:** 10.1186/s13104-019-4759-1

**Published:** 2019-11-28

**Authors:** Awrajaw Dessie, Tesfay Gebregzabher Gebrehiwot, Berihu Kiros, Sintayehu Daba Wami, Daniel Haile Chercos

**Affiliations:** 1 Department of Environmental and Occupational Health and Safety, Institute of Public Health, University of Gondar, Gondar, Ethiopia; 2 School of Public Health, College of Health Science, Mekelle University, Mekelle, Ethiopia

**Keywords:** Intestinal parasitic infections, Children, Prevalence, Polyparasitism, School

## Abstract

**Objective:**

This study aimed to determine the prevalence of intestinal parasitic infections and associated factors among school-age children in Sebeya primary school, northern Ethiopia, 2017.

**Results:**

The prevalence of intestinal parasites in school-age children was (29.9%). A total of six parasites were detected in this study. *E. histolytica*/*dispar* 19.43% (82/422) and *G. lamblia* 8.29% (35/422) were the predominant ones. Unclean fingernail (AOR = 1.72), defecating in the open field (AOR = 2.82), and being barefooted (AOR = 1.72) were the determinant factors for intestinal parasitic infections. Frequently washing hands reduced the chance of infections by 68%. Furthermore, children in grade 1–4 and 5–6 had higher odds developing the infections than those in grade 7–8 (AOR = 8.21 and AOR = 2.50, respectively).

## Introduction

Helminths and protozoa species, causing intestinal parasitic infections, are endemic worldwide. It is considered the pressing public health issues, predominantly in tropical and subtropical countries. About 3.5 billion individuals are impacted, of which and 450 million people suffering from the diseases [1]. In resource limited parts of the world, the problem of intestinal parasite diseases is deep [2]. The intestinal parasites are extremely common in crowded or unhealthy circumstances such as refuse heaps, gutters and wastewater units in and around people’s dwelling and living circumstances [3].

Children are disproportionately at risk for the infections, amongst others, owing to their increased nutritional requirements and less developed immune systems [4]. Intestinal parasite diseases linked with intestinal bleeding, nutrient malabsorption, nutrient deficiency, and cell and tissue destruction, anemia, intestinal obstruction, and mental and physical development retardation among children. Overall, these results in delayed growth, decreased mental development, school absenteeism, low academic achievement, prone to malnutrition and infection [5–7].

In Ethiopia, infections with intestinal parasites are toping the morbidity list in different health facilities. *A. lumbricoides*, *T. trichiura, H. nana, histolytica/dispar,* and *S. mansoni* are highly spread in the country [8–11]. There are, however, still several locations for which epidemiological data, including the research region, is not accessible. Poor environmental and personal hygiene, food contamination, unsafe drinking water, and improper management of urine and faeces led to the occurrence of the infection [12]. Poverty, analphabetism, bad sanitation, water inaccessibility, and warm and humid tropical climate have also been correlated with parasitic intestinal diseases in Ethiopia [10].

Determining the status of intestinal parasitic diseases and its correlates is crucial not only to formulate appropriate control strategies but also to envisage the risk to considered groups. Existing knowledge indicated the problem is differing from location to location based on the different variables enlisted in Ethiopia and there is scanty of information in the study area. This study, therefore, aimed to determine the prevalence and risk factors of intestinal parasites among schoolchildren in Northern Ethiopia.

## Main text

### Methods

#### Study setting and period

The study was conducted in Sebeya primary school from January to February 2017. It is located in Glomekeda district, northern Ethiopia, which 915 km far from Addis Ababa, the capital city of Ethiopia. The school is located in altitude of 2528 m above sea level and geographically it is located 14° 28′ 14.15′′ N latitude and 39° 29′ 40.5′′ E longitude.

#### Study design

Institution based cross-sectional study was employed.

#### Study population

School-age children from grade 1 to 8 who were available during the period of data collection, whose parents/guardians have given assent and who agreed to participate were included.

#### Sample size determination and sampling technique

The sample size was determined using a single population proportion formula, considering the proportion of intestinal parasitic infection 47.1% [13], a margin of error 5%, confidence interval of 95%, as assumptions. The final sample size was 422 including a 10% non-response rate students. Samples were selected by simple random sampling technique. Proportional allocation was made to select the study subjects from each grade level based on the number of students at each grade level.

#### Operational definitions

*Intestinal parasitic infection (IPs):* The participant was recorded positive for IPs if the stool sample examined by microscopy becomes positive to cyst or trophozoite of any parasite.

*Washing hands at critical times:* Washing hands after visiting the toilet, before a meal, after touching the bottom of the child, after touching animal pets, before food preparation.

*Knowledge:* Participants were asked to answer 10 knowledge questions about transmission and prevention methods of intestinal parasitic infection. Graded as having “Good knowledge” if they had answered correctly (≥ 80%) 8–10 questions, medium if they had answered (50–70%) 5–7 questions, and < 5 questions as “Poor knowledge”.

#### Data collection tools and procedure

To collect appropriate data from each student, a structured questionnaire was prepared. Parents and guardians of the chosen learners were traced and interviewed for socio-demographic variables, environmental factors, and hygienic behavior. Meanwhile, data collectors verified the accessibility of the bathroom and the fingernail cleanliness of each student.

#### Stool sample collection and processing

Two gram of new fecal samples were gathered from children and put in separately labeled containers of smooth plastic stools. The nature of the stool sample was observed and the date of sampling was also noted. The specimens were preserved with 10% formalin and transported to the Mekelle University Medical Parasitology Laboratory. To identify parasitic organisms, a method of formol-ether concentration was employed as described by the standard method [14].

#### Quality control

Data collectors and supervisors have been trained on the methods of data collection. On the spot checking of the completeness of the data was done. The stool specimen examination was done following standard laboratory procedures and few randomly selected samples were re-checked for verification.

#### Data processing and analysis

Data were entered into EPI Info 7 and exported to SPSS 20 for analysis. Mean, frequency and percentage were used for description. A binary logistic regression model was used to identify significantly associated variables at a *p* value < 0.05. During bivariate analysis, variables with a *p*-value < 0.2 were candidates for multivariable logistic regression for the final model. Hosmer and Lemeshow goodness-of-fit test were performed.

### Results

#### Socio-demographic characteristics

This research involved a total of 422 school children, which yields 100% response rate. About 227 (53.79%) were females. The mean (± SD) age of study participants was 11.5 (± 2.31) years. Half of the parents (50.24%) receive less than 500 ETB of monthly income (Additional file 1: Table S1).

#### Environmental and behavioral characteristics

Two-thirds of children’s parents (63.6%) collected water from well water and 258 (61.1%) washing their hands at critical moments. Two hundred and forty-three (57.6%) of children frequently brushed their teeth and 183 (43.8%) clean their fingernail. More than two-thirds of children (65.4%) always had to wear shoes. Two hundred and thirty-nine (56.6%) children had little knowledge on prevention and control of intestinal parasite infection (Table 1).

**Table 1:** Environmental and behavioral characteristics of Sebeya primary school children, January to February, 2017 (n = 422)

Variables	Frequency (n)	Percent (%)
Type of water source		
Pipe	58	13.7
Spring	96	22.7
Hand dug well	268	63.6
Open field defecation		
Yes	35	8.3
No	387	91.7
Washing hands at critical times		
Yes	258	61.1
No	164	38.9
Frequency of taking bath		
Once a week	84	19.9
Once in a month	61	14.5
Twice a month	277	65.6
Brushing teeth regularly		
Yes	243	57.6
No	179	42.4
Frequency of washing cloth		
Once a week	42	10.0
Twice a month	134	31.8
Once a month	246	58.2
Clean finger nail		
Yes	185	43.8
No	237	56.2
Shoe wearing habit		
Regular	276	65.4
Irregular	146	34.6
Knowledge		
Knowledgeable	103	24.4
Not knowledgeable	319	75.6

#### Prevalence of the intestinal parasite infection

The prevalence of intestinal parasite infection was 29.9% (95% CI 27.7–32.1%). About 31.3% (61/195) of males were infected with at least one parasite and 28.6% (65/227) of females had the infection. The rate of single, double, and triple parasite infections was 73.02% (92/126), 20.63% (26/126), and 6.35% (8/126), respectively. A total of six parasites were detected and *E. histolytica/dispar* 19.43% (82/422) and *G. lamblia* 8.29% (35/422) were the predominant ones (Table 2).

**Table 2:** Distribution of intestinal parasites species among Sebeya primary school children, January to February, 2017 (n = 422)

Type of intestinal parasite	Number of male +ve (%)	Number of female +ve (%)	Total number of +ve (%)
Single infection (n = 92)			
*E. histolytica/dispar*	26 (6.2)	24 (5.7)	50 (11.8)
*G. lamblia*	8 (1.9)	12 (2.8)	20 (4.7)
*A. lumbricoides*	8 (1.9)	6 (1.4)	14 (3.3)
*H. nana*	2 (0.5)	1 (0.2)	3 (0.7)
Hookworm	1 (0.2)	2 (0.5)	3 (0.7)
*T. trichiura*	1 (0.2)	1 (0.2)	2 (0.5)
Double infection (n = 26)			
*E. histolytica/dispar* and *G.lamblia*	6 (1.4)	4 (0.9)	10 (2.4)
*E. histolytica/dispar* and *A. lumbricoides*	4 (0.9)	3 (0.7)	7 (1.7)
*E. histolytica/dispar* and *H. nana*	2 (0.5)	5 (1.2)	7 (1.7)
*G. lamblia* and *A. lumbricoides*	0 (0.0)	2 (0.5)	2 (0.5)
Triple infection (n = 8)			
*E. histolytica/dispar, G. lamblia and* *H. nana*	2 (0.5)	1 (0.2)	3 (0.7)
*E. histolytica/dispar, H. nana* and *Hookworm*	0 (0.0)	3 (0.7)	3 (0.7)
*E. histolytica/dispar, Hookworm* and *T. trichiura*	1 (0.2)	1 (0.2)	2 (0.5)
Total	61 (14.5)	65 (15.4)	126 (29.9)

#### Factors associated with intestinal parasitic infection

In multivariable analysis; grade level, father occupation, clean fingernail, open field defecation, hand washing at critical times, and shoe wearing habit were discovered to be statistically associated with parasitic intestinal diseases (p-value < 0.05) (Table 3).

**Table 3:** Multivariate analysis for factors potentially associated with Intestinal parasite infection among Sebeya school children, January to February, 2017

Risk factor	Status of intestinal parasitic infections		COR (95% CI)	AOR (95% CI)
	Positive, n (%)	Negative, n (%)		
Grade level				
1–4	90 (50.6)	88 (49.4)	9.29 (4.78, 18.06)**	8.21 (3.88, 17.48)**
5–6	24 (19.5)	99 (80.5)	2.20 (1.05, 4.64)**	2.51 (1.14, 5.540)*
7–8	12 (9.9)	109 (90.1)	1.00	1.00
Father occupation				
Farmer	88 (28.5)	221 (71.5)	1.25 (0.66, 2.35)	1.26 (0.62, 2.53)
Daily labourer	16 (51.6)	15 (48.4)	3.34 (1.34, 8.33)**	2.96 (1.07, 8.18)*
Trader	7 (35.0)	13 (65.0)	1.69 (0.57, 5.00)	1.28 (0.38, 4.22)
Civil servant	15 (24.2)	47 (75.8)	1.00	1.00
Clean finger nail				
Yes	43 (23.2)	142 (76.8)	1.00	1.00
No	83 (35.0)	154 (65.0)	1.78 (1.15, 2.74)**	1.72 (1.04, 2.85)**
Open field defecation				
Yes	21 (60.0)	14 (40.0)	4.03 (1.98, 8.21)**	2.82 (1.21, 7.45)**
No	105 (27.1)	282 (72.9)	1.00	1.00
Washing hands at critical times				
Yes	71 (27.5)	187 (72.5)	0.45 (0.29, 0.69)**	0.32 (0.16, 0.65)*
No	55 (33.5)	109 (66.5)	1.00	1.00
Shoe wearing habit				
Regular	57 (31.5)	124 (68.5)	1.00	1.00
Irregular	82 (32.3)	172 (67.7)	3.44 (2.22, 5.33)**	1.72 (1.02, 2.91)**

1.00 = Reference, *significant at p value < 0.05, **significant at p value < 0.001

According to this study, children in grade 1–4 and 5–6 were 8.21 and 2.5 times more likely than their counterparts to have intestinal parasitic diseases [AOR = 8.21, 95% CI 3.88, 17.47, and AOR = 2.50, 95% CI 1.14, 5.54]. Children whose father’s work was a daily worker were 2.96 times more likely than children whose father’s job was a civil servant to develop intestinal parasitic diseases [AOR = 2.96, 95% CI 1.07, 8.18].

Children with unclean fingernails were 1.72 times more probable than children with clean fingernails to develop parasitic intestinal infections [AOR = 1.72, 95% CI 1.04, 2.85]. On the other side, children who were washing hands at critical moments were 68% less probable than their counterparts to develop intestinal parasite infection [AOR = 0.32, 95% CI 0.16, 0.65]. Children who defecate in the open field were 2.82 times higher odds of developing intestinal parasite infections compared to children who did not practice open field defecation [AOR = 2.82, 95% CI 1.21, 7.45]. Besides, the prevalence of intestinal parasite diseases was 72% greater in children with uneven shoe habit [AOR = 1.72, 95% CI 1.02, 2.91].

## Discussion

The purpose of this research was to evaluate the prevalence of parasitic intestinal diseases among school-age children. In the current research, the prevalence of intestinal parasite diseases was discovered to be 29.9%. This finding is comparable with other studies that reported 27.2% in Babile [9], and 29.3% in Mekelle [15]. On the other hand, the prevalence was greater than the study conducted in Tilili, Ethiopia, which was reported to be 26.2% [11], and in Butajira, Ethiopia, which was 23.3% [16]. To the contrary, higher prevalence of intestinal parasites disease was found from 58 to 83.8% in different towns of Ethiopia [17–20]. Similarly, a higher prevalence of the disease compared to the current finding was reported in sub-Saharan Africa; e.g. 48.7% in Tanzania [23], and 90.4% in Central Sudan [24]. The distinction in parasitological diagnostic techniques used, level of environmental sanitation, source of drinking water, family education and personal hygiene may be correlated with the discrepancy [21].

Grade level of school children was found to be a significant predictor of intestinal parasitic infections; lower-grade school children had higher probability of developing the infections when compared to their counterparts. Children of lesser grade tend to play in the open field and generally touch their mouth with their dirty fingers may explain the result. Their understanding of disease prevention and control mechanisms could be small, further predisposing to diseases with parasites. The finding is consistent with comparable research in Ethiopia showing that children in lower grade had a greater prevalence of intestinal parasite diseases [22, 23].

Interventions in public health including access to safely managed drinking water, community health education, food hygiene, functional sanitation systems are key for long-term intestinal parasite control [21, 24–26]. In this research, the main modifiable predictors’ hygiene behavior such as the cleanliness of fingernail and not handwashing, and open defecation. Chances of infection with intestinal parasites have risen by 72% among children with unclean fingernails compared to children with smooth fingernails, which is consistent with other research [21, 23, 27, 28]. Because the fecal–oral path is the primary pathway for the spread of parasite diseases, unclean fingernails can increase the incidence and intensity of intestinal parasite diseases [27]. Because their habitat is unclean fingers, the life-cycle of soil-borne intestinal parasites can cause direct feco-oral transmission of intestinal parasites.

This research showed that the risk of intestinal parasite infection among children who commonly washed their hands at critical moments was lowered by 68% compared to children who did not wash. This finding is comparable with the study conducted in different parts of Ethiopian [21, 23, 27]. This can be because children get germs when they touch infected items, which increases the probability of disease. Hand washing is, therefore, the most efficient way to prevent infection from spreading [27, 29, 30]. Similarly, among children who used protective shoes irregularly compared to their counterparts, the chance of affected by intestinal parasite infections increased by 72%. The finding is in agreement with comparable research in developing nations [17, 21, 28]. Possible explanations of shoe-wearing habit link and enhanced infection with parasites may be due to enhanced potential for infection with hookworm. Lack of protective shoes is known to boost the likelihood of infection with hookworms [21].

This study revealed that there was a higher probability of parasite illnesses among children defecating in the open field. This was in agreement with other studies [31, 32]. The possible explanation might be improper management of human feaces led to disease-causing pathogens, particularly infectious eggs and helminth larvae would litter the atmosphere. As these illnesses are transferred through the fecal–oral route or direct skin penetration, the risk of infection with such environmental contamination will increase [25, 26].

## Conclusion

In the studied area, the prevalence of intestinal parasite diseases among school-age children was high. Infection with polyparasitism was also a concern. Improving socioeconomic status, enhancing sanitation services, instilling health education and encouraging methods to maintain personal hygiene, constantly developing shoe habit can be excellent strategies for controlling these infections in the region. It is suggested to create a school-based understanding of how intestinal parasites are transmitted.

## Limitations

The study’s main limitation was that we did not measure the intensity of the parasite infection, which might better illustrate the degree of infection in the study area. Due to the lack of laboratory equipment, opportunistic infections were not evaluated.

## Supplementary information

**Additional file 1: Table S1**. Socio demographic characteristics of Sebeya primary school children, January to February, 2017 (n = 422).

## Data Availability

Data will be made available upon request the primary author.

## Abbreviations

AOR: adjusted odds ratio
CI: confidence interval
COR: crude odds ratio
SD: standard deviation
SPSS: statistical package for social sciences
