# Supplementary material for: Intestinal parasitic infections and determinant factors among school-age children in Ethiopia: a cross-sectional study
Source: BMC Res Notes. 2019 Nov 28;12:777. doi: 10.1186/s13104-019-4759-1 (PMC6883565; doi:10.1186/s13104-019-4759-1)
Supplement: Supplementary file 1 — Additional file 1: Table S1. Socio demographic characteristics of Sebeya primary school children, January to February, 2017 (n = 422). [file 13104_2019_4759_MOESM1_ESM.docx]

Additional file 1:Table S1. Socio demographic characteristics of Sebeya primary school children, January to February, 2017 (n=422).

| Variables | Frequency (n) | Percent (%) |
| --- | --- | --- |
| Sex |  |  |
| Male | 195 | 46.21 |
| Female | 227 | 53.79 |
| Age in years |  |  |
| 7-9 | 91 | 21.56 |
| 10-12 | 145 | 34.36 |
| 13-15 | 186 | 44.08 |
| Grade level |  |  |
| Grade 1-4 | 178 | 42.18 |
| Grade 5-6 | 123 | 29.15 |
| Grade 7-8 | 121 | 28.67 |
| Religion |  |  |
| Orthodox | 402 | 95.26 |
| Catholic | 12 | 2.84 |
| Muslim | 8 | 1.90 |
| Mother's education level |  |  |
| Not able to read and write | 190 | 45.02 |
| Grade 1-8 | 101 | 23.93 |
| Grade 9-12 | 111 | 26.30 |
| Higher education | 20 | 4.74 |
| Father's occupation |  |  |
| Farmer | 309 | 73.22 |
| Daily labourer | 31 | 7.35 |
| Trader | 20 | 4.74 |
| Civil servant | 62 | 14.69 |
| Parent's monthly income (ETB) |  |  |
| < 500 | 212 | 50.24 |
| 500-1500 | 164 | 38.86 |
| > 1500 | 46 | 10.90 |

**NB: ETB = Ethiopian Birr**
